# Tackling burnout in Australian doctors by blending a web-based cognitive-behavioural therapy program with telehealth psychological support – protocol for a three-arm randomised-controlled trial

**DOI:** 10.1016/j.conctc.2025.101514

**Published:** 2025-06-30

**Authors:** M.J. Coleshill, M.J. Black, K. Luck, K. Willis, N. Smallwood, H. Stephens, T. Gillings, L. Fraser, M. Putland, L. Kampel, A.M. Martin, N.F. Praharso, A.D. Joffe, S. Harvey, P.A. Baldwin

**Affiliations:** a Black Dog Institute, Sydney, Australia; b UNSW Medicine & Health, University of New South Wales, Sydney, Australia; c School of Psychology, University of New South Wales, Sydney, Australia; d Institute for Health & Sport, Victoria University, Melbourne, Australia; e School of Translational Medicine, Monash University, Melbourne, Australia; f Department of Respiratory & Sleep Medicine, Alfred Hospital, Melbourne, Australia; g Department of Emergency Medicine, Royal Melbourne Hospital, Melbourne, Australia; h Department of Critical Care, Faculty of Medicine, University of Melbourne, Melbourne, Australia

**Keywords:** Blended care, Digital, Mental health, Burnout, Doctors, Health professionals

## Abstract

**Background:**

Burnout has received limited attention in treatment programs, despite high prevalence among health professionals and the threat burnout places upon the mental health and the long-term sustainability of the Australian healthcare system. As part of The Essential Network (TEN), a blended care mental health support service for Australian health professionals, we developed Navigating Burnout – a digital cognitive-behavioural therapy program for health professional burnout. This three-arm randomised-controlled trial (RCT) will examine the effectiveness, acceptability, and cost-effectiveness of Navigating Burnout in both blended care and digital formats in reducing burnout among doctors.

**Methods:**

Doctors (n = 207) with burnout will be randomised to (1) a blended version of Navigating Burnout combining digital resources with five fortnightly telehealth sessions with a clinical psychologist, (2) a digital-only version of Navigating Burnout, or (3) self-care psychoeducation as an active attention control. Burnout, psychosocial wellbeing, workforce engagement and attrition, and service acceptability will be measured at baseline, post-treatment, and 3 months post-treatment.

**Results:**

At 3 months post-treatment, we hypothesise reductions in burnout across both treatment arms, with the strongest effect in the blended care arm. Similar trends are expected for psychosocial and occupational outcomes. High service acceptability across both blended care and digital-only versions of Navigating Burnout is also anticipated.

**Conclusions:**

With this evidence, Navigating Burnout may be incorporated into TEN's person-to-person components. Further, by demonstrating the effectiveness of blended care for burnout, Navigating Burnout may provide a crucially needed service for Australian doctors and replicable model of care for other organisations and support services.

## Highlights

• Health professional burnout has received limited attention in treatment programs.
• Navigating Burnout is a digital cognitive-behavioural therapy program for burnout.
• This trial compares blended care and self-guided versions with a self-care control.
• 207 doctors will engage with treatment for 10 weeks, with a 3-month follow-up.
• Blended care Navigating Burnout is expected to reduce burnout among doctors.

## 1 Introduction

The healthcare industry places significant demands on doctors, including protracted working hours in highly stressful conditions [1] alongside understaffing [2] and a lack of job control [3]. The COVID-19 pandemic intensified these pressures, leading to moderate-to-severe burnout in approximately 50% of health professionals, with doctors more likely to experience physical and emotional exhaustion than their non-medical colleagues [4].

Burnout is a work-related syndrome characterised by emotional exhaustion, disengagement from work, and a reduced sense of accomplishment [5]. Burnout is not currently listed in the Diagnostic and Statistical Manual of Mental Disorders as a diagnosable mental disorder, and there is an ongoing debate as to whether burnout constitutes a type of depression or a unique condition [6,7]. Nevertheless, the syndromic characteristics of burnout have been shown to jeopardise doctors’ mental health [8], reduce professionalism in healthcare [9], increase workplace attrition [10], and compromise patient safety [9,11]. Indeed, a recent meta-analysis found that high levels of burnout among doctors was associated with a doubling of patient safety incidents, as well as a threefold increase in turnover intention [9]. These outcomes pose significant risks to the effectiveness and sustainability of the healthcare system for the public benefit.

Despite the wide prevalence and known risks of burnout in doctors, almost no established programs exist to help address burnout in doctors or health professionals more broadly. While there is a growing local [12] and international [5] consensus that burnout in medicine has systemic causes that require systemic interventions, most current research focuses on supporting individuals. Several meta-analyses have found mixed support for the efficacy of the limited available interventions in addressing burnout, in part due to high heterogeneity in definition and measurement of burnout, intervention content, and quality of trial design [[13], [14], [15]]. Despite this heterogeneity, several studies have found support for web-based cognitive-behavioural therapy (CBT) as a promising and scalable treatment option [16,17].

In 2020, the Black Dog Institute (BDI) launched The Essential Network (TEN), funded as part of the Australian Government's response to COVID-19 [18]. TEN is a blended care mental health support service designed by-and-for health professionals that combines digital and person-to-person care. TEN provides healthcare professionals with access to an online digital gateway which features tools such as a digital mental health check-up and evidence-based programs and resources tailored to the needs of health professionals. Digital services are complemented by connection to person-to-person care with up to with up to five free telehealth sessions with a clinical psychologist or psychiatrist through the BDI Clinical Service.

Although evaluations found that the initial TEN program provided effective support for psychological stress management, the initial program did not adequately address the more prominent complaint of burnout [19,20]. Based on this insight, as well as the virtual absence of tailored treatment programs for burnout in health professionals, we developed a first-of-kind digital program called “Navigating Burnout” to expand the TEN program. Navigating Burnout is a novel self-guided digital CBT and acceptance and commitment therapy (ACT) program tailored to the needs of health professionals experiencing, or at risk of, burnout. Upon successful evaluation, Navigating Burnout will be fully integrated into the TEN program.

This randomised-controlled trial (RCT) aims to establish evidence on the effectiveness, acceptability, and cost-effectiveness of Navigating Burnout across blended and self-guided modes of delivery. Such evidence will inform the integration of Navigating Burnout into TEN's person-to-person care to better support health professionals experiencing burnout, as well as highlight the efficacy of Navigating Burnout as a scalable digital program to mitigate burnout among health professionals.

## 2 Methods

### 2.1 Trial design

We will conduct a three-arm RCT to compare the effectiveness, acceptability, and cost-effectiveness of Navigating Burnout in reducing burnout among doctors across three delivery modalities.

Medical doctors (n = 207) will be randomly allocated to receive either [1] a blended version of the program combining the digital Navigating Burnout resources with five fortnightly telehealth sessions with a clinical psychologist [2]; a digital-only, self-guided version of the Navigating Burnout program; or [3] an active attention control where participants will be provided with digital self-care psychoeducation via email each fortnight. Across all conditions the treatment phase across will last 10 weeks.

**Fig. 1:**
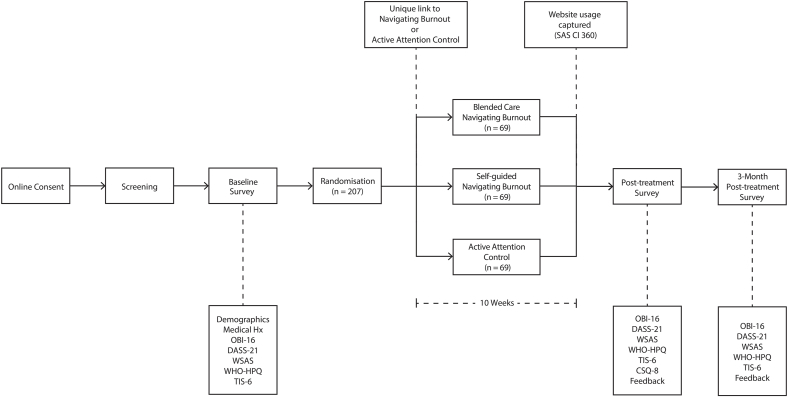
Schematic diagram of trial design. Figure legend: Oldenburg Burnout Inventory (OLI-16), Depression Anxiety Stress Scale (DASS-21), Work and Social Adjustment Scale (WSAS), World Health Organisation Health and Work Performance Questionnaire (WHO-HPQ), Turnover Intention Scale-6 (TIS-6), and Client Satisfaction Questionnaire (CSQ-8).

We will measure self-reported burnout, psychosocial wellbeing, workforce engagement, and workforce attrition intentions before treatment, immediately after treatment, and 3 months after completing treatment (see Fig. 1 for trial design). This design will enable us to examine the effectiveness and acceptability of blended care Navigating Burnout compared to self-guided Navigating Burnout, as well as to examine the effectiveness of self-guided Navigating Burnout relative to an appropriate control.

### 2.2 Participants

Individuals will be eligible to participate in the trial if they satisfy all of the following criteria.

• registered doctor currently practising in Australia;
• able to read and speak English fluently;
• current experience of self-reported burnout;
• able to access the internet in a private location on a device suitable for viewing websites and attending telehealth consultations; and
• able to attend telehealth consultations during Monday to Friday business hours (9.00–17.00).

Individuals will be excluded from trial participation if they satisfy any one or more of the following criteria.

• current diagnosis of severe mental illness that requires more specialist support (e.g., bipolar disorder, schizophrenia, etc.);
• current engagement with person-to-person psychological therapy (e.g. cognitive-behavioural therapy);
• DASS-21 symptom scores in the Severe or Extremely Severe ranges for anxiety or depression during screening; or
• report suicidal ideation or problematic alcohol or drug use during screening.

### 2.3 Interventions

**Fig. 2:**
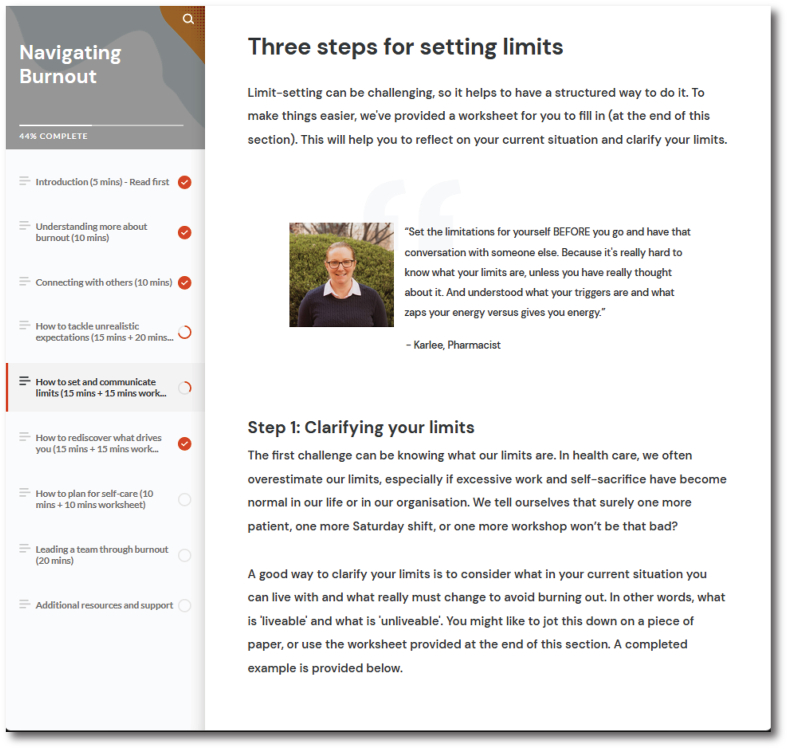
Example screenshot from Navigating Burnout.

Navigating Burnout is a digital psychotherapeutic program that includes intervention components derived from evidence-based cognitive-behavioural and acceptance and commitment therapies that are delivered across seven topics [1]: understanding burnout [2]; increasing social connections [3]; managing unrealistic expectations [4]; setting limits [5]; valued living [6]; self-care; and [7] a module for managers detailing evidence-based strategies for improving workplace mental health (see Fig. 2). Each topic contains lived experience videos, behavioural strategies, and CBT worksheets.

In the blended care arm, a clinical psychologist from the BDI Clinical Service will work through the Navigating Burnout content with participants over 10 weeks in five fortnightly 1-hour consultations. Clinical psychologists will follow a treatment manual mirroring the content of Navigating Burnout, with guidance on integrating digital content into session activities. Session 1 covers understanding burnout and self-care, session 2 covers unrealistic expectations, session 3 covers setting limits, session 4 covers valued living, and session 5 covers increasing social connections and information for managers. Session 1 is fixed and the same for all participants, whereas sessions 2–5 occur in an order determined by the participant and their clinical psychologist. Clinical psychologists will be offered a training program comprising [1]: a comprehensive treatment manual summarising the study protocol, the digital content, and session plans with suggested approaches to delivering the program's CBT strategies [2]; a 90-min protocol training session delivered by PB covering the design and purpose of the study, how to use the training manual, how to embed the digital content in a standard telehealth consultation, and roleplays of case examples; and [3] optional monthly 60-min clinical supervision sessions with PB. Clinicians will have discretion to tailor consultations to participant needs as long as (a) the clinical focus remains burnout; and (b) the program content is delivered in full across the five consultations. Participants will also have independent access to the digital version of Navigating Burnout over the 10 weeks between consultations and be directed by their clinical psychologist to specific components of the program in order to support awareness and management of burnout. During the 10-week treatment period, clinical psychologists providing care in the blended arm will provide immediate risk assessment and intervention for any participant expressing intent to self-harm or suicide and report any concerns related to suicide or self-harm attempts to PB.

In the self-guided arm, participants will be emailed a link to Navigating Burnout to engage with independently over 10 weeks. While participants will have access to the entirety of Navigating Burnout from onset, progress will be supported by fortnightly structured reminders that encourage participants to continue working through the course.

### 2.4 Control

In the control condition, participants will receive an active attention control comprising of fortnightly emails containing links to generic digital self-care psychoeducation materials. The digital self-care psychoeducation was based on the self-care component of Navigating Burnout, expanded to include additional generic self-care content. The email cadence was chosen to align with the fortnightly structure of reminders in the self-guided arm (i.e., emails will provide participants with information to work through fortnightly). While self-care is included in Navigating Burnout for the purpose of maintaining or improving general mental well-being, previous research has found that self-care alone does not produce a measurable impact on burnout outcomes [21]. Thus, self-care materials were chosen as a suitable attention control that is not expected to treat burnout.

### 2.5 Recruitment, screening, and consent

The research team will work with the BDI marketing and communications team to recruit participants by providing advertisements on social media (i.e. Facebook, Instagram) and emailing health professionals on an internal BDI mailing list of health professionals interested in research participation.

Additionally, independent industry organisations Barwon Health, Peninsula Health, Royal Melbourne Hospital, and La Trobe Regional Hospital will be invited to distribute study advertisements to their medical employee networks. Potential participants will be provided with a REDCap link to the Patient Information Statement and Consent Form (PISCF) where they can view more information about the trial. The contact details for the Coordinating Principal Investigator will be provided in recruitment materials and the PISCF to offer potential participants the opportunity to ask questions and seek further information.

Following consent, an online screening survey will be completed through REDCap. It will ask potential participants to confirm the inclusion criteria, as well as complete a modified version of the DASS-21 to confirm the absence of exclusion criteria. The modified DASS-21 includes two risk-related items addressing suicidal ideation (“I had thoughts that I would be better off dead, or thoughts of hurting myself in some way”) and problematic use of alcohol or drugs (“I found it difficult to control my consumption of alcohol or other drugs”) rated on the same 0–3 scale as the other questions in the DASS-21 ranging from “Did not apply to me at all” to “Applied to me very much or most of the time”. Potential participants are ineligible to participate if they report DASS-21 symptom scores in the Severe or Extremely Severe ranges for anxiety (DASS-21 anxiety subscale score >7) or depression (DASS-21 depression subscale score >10) or report suicidal ideation or problematic alcohol or drug use (score >1 on respective questions). If a person does not meet the inclusion criteria, they will be notified by a message within the online survey. Branching logic will be used to present the participant with the relevant reason(s) that they were not eligible and, where relevant, provide links to crisis services and/or TEN where they may find support. If a person reports suicidal ideation or problematic alcohol or drug use during screening (rating of “Often” or “Almost Always” on screening items addressing these criteria) a clinical psychologist (PB or MB) will contact the participant directly by email to direct the person to appropriate supports.

To confirm that all participants are registered doctors following successful online screening and prior to data collection, potential participants' name, postcode, and profession will be checked against the Australian Health Practitioner Regulation Agency (AHPRA) database – a publicly available list of registered health professionals in Australia. In the event that a participant's registration cannot be verified, they will be contacted by email for clarification.

### 2.6 Randomisation

Eligible participants will be randomly allocated to the blended care, self-guided, or active attention control arm using a 1:1:1 ratio. Randomisation will be completed following completion of baseline outcomes.

A randomisation list in a Microsoft Excel file will be prepared in advance using a random number generator using block sizes of 6, 9, and 12. When a participant is ready to be randomised, the list holder will be contacted, and the allocation provided. Participants will be informed which treatment they have been randomly assigned via email.

### 2.7 Blinding

By nature of the interventions being examined, complete single blinding of participants to intervention allocation is not possible. Instead, to improve allocation concealment participants will be informed that the trial is examining a bespoke burnout intervention for health professionals containing both person-to-person and self-guided digital components and that they may be allocated to receive one or all these components. This procedure will serve to ensure that allocation concealment between the self-guided Navigating Burnout and active attention control is maintained.

As Navigating Burnout is currently publicly available through TEN without registration, there is a risk that participants in the active control arm could access Navigating Burnout outside the scope of the trial. As such, references to TEN and Navigating Burnout will be limited in recruitment materials and the PISCF to reduce the risk of contamination.

### 2.8 Procedure

Eligible participants will complete baseline surveys (demographics and psychometrics) before random allocation to self-guided, blended, or active attention control. All arms will access their allocated treatment over 10 weeks. Participants will complete follow-up surveys (psychometrics, service acceptability questions) immediately post-treatment, and again three months post-treatment to assess maintenance of therapeutic effects.

Following completion of the 3 months post-treatment survey, or once participants are considered lost to follow-up at the 3 months post-treatment timepoint, participants allocated to the self-guided or active attention control will be provided with information about TEN, including information on how to access the self-guided Navigating Burnout and/or person-to-person care.

Participants will complete 6 psychometric questionnaires: Oldenburg Burnout Inventory (OLBI; burnout) [22], Depression Anxiety Stress Scale (DASS-21; depression and anxiety) [23], Work and Social Adjustment Scale (WSAS; psychosocial functioning) [24], World Health Organisation Health and Work Performance Questionnaire (WHO-HPQ; workforce participation) [25], Turnover Intention Scale-6 (TIS-6; workforce attrition) [26], and Client Satisfaction Questionnaire (CSQ-8; intervention acceptability) [27]. To further assess acceptability, bespoke questions regarding the self-reported usage and acceptability of Navigating Burnout will also be employed, alongside open-ended questions about participants’ experiences with their allocated treatment and their experiences of burnout.

The OLBI, DASS-21, WSAS, WHO-HPQ, and TIS-6 will be completed at baseline, post-treatment, and 3 months post-treatment, while the CSQ-8 and service acceptability questions will only be completed at the post-treatment timepoint. See Table 1 for an overview of measures employed across trial timepoints. Regular safety audits (2–3 times per week) of the post-treatment and 3 months post-treatment surveys will be conducted, and any responses indicative of distress or suicidal ideation will be reported to PB. If a participant consented to a check-in call related to indicators of distress or suicidal ideation, a clinical psychologist (PB or MB) will contact the participant immediately.

**Table 1:** Measures employed across trial timepoints.

Measure	Baseline	Post-treatment	3 months post-treatment

Demographics	X		
OLBI	X	X	X
DASS-21	X	X	X
WSAS	X	X	X
WHO-HPQ	X	X	X
*TIS-6*	X	X	X
CSQ-8		X	
Self-reported service usage		X	X
Objective service usage		X	X
Open-ended service acceptability		X	
Open-ended burnout	X	X	X

In the blended care arm, participants will be asked to complete an additional OLBI and DASS-21 if their first consultation is arranged for more than three weeks after their baseline survey is completed. This measure will be provided to participants two weeks before their first consultation date. This will ensure the primary outcome remains valid despite delays to treatment start in the blended care arm (e.g., due to treating clinicians being at capacity or participants' availability), as well as provide treating clinicians with updated data on participants’ current mental health.

To aide participant retention, participants will be reimbursed for their participation by means of a $25 digital gift card sent via the GiftPay system to their nominated email at the completion of each measurement point (i.e. baseline, post-treatment, and 3 months post-treatment). To minimise attrition, participants who do not return survey data will receive up to 3 automated survey reminders via email every 2 days from the initial survey invitation date. Those who do not return survey data after 2 weeks past the final automated reminder will receive a final reminder from a member of the research team sent from the trial email account. To further aide engagement in the in the blended care arm, participants will liaise with a member of the research team or patient care coordinator from the Black Dog Institute Clinical Service regarding consultations and attendance (e.g. organising of consultations in a piecemeal manner when a participant cannot commit to all 5 sessions from initial contact due to scheduling issues, rescheduling of cancelled appointments, etc).

### 2.9 Primary outcome

The primary outcome will be self-reported burnout, as measured by the OLBI, with the primary endpoint being 3 months post-treatment.

### 2.10 Secondary outcomes

The secondary outcomes will be.

• burnout (OLBI) at post-treatment
• self-reported anxiety, depression, and stress (DASS-21) at post-treatment and 3 months post-treatment;
• psychosocial functioning (WSAS) at post-treatment and 3 months post-treatment;
• workforce participation (WHO-HPQ) at post-treatment and 3 months post-treatment;
• workforce attrition intentions (TIS-6) at post-treatment and 3 months post-treatment;
• service acceptability (CSQ-8) at post-treatment.

### 2.11 Treatment fidelity and service usage

To assess fidelity of telehealth sessions in the blended care arm, the treating clinical psychologist will complete a survey after each session answering 3 questions [1]: *“Which topics from the clinical manual were covered in this session?“*, recorded as a single item response to a list of the available Navigating Burnout modules [2]; *“How closely did you follow the suggested structure for Topic X?”* where “X” refers to the module selected in question one, recorded as a single item response to a list of three options of “Not at all”, “Mostly” or “Completely”; and [3] *“Did the session identify any participant risks that require escalation?“*, recorded as a single item response to two options of “Yes” or “No”. Clinicians were also offered an optional free text field to record any session details they deemed relevant to treatment fidelity. Quantitative data will be analysed to assess protocol coverage, protocol adherence, and risk frequency. Qualitative data will be reviewed for additional threats to fidelity.

Navigating Burnout and Black Dog Institute website analytics are recorded automatically by the SAS CI360 platform for service evaluation purposes. As Navigating Burnout is publicly available without registration, participants in the self-guided arm will be provided with a unique link to Navigating Burnout containing their record ID on REDCap (e.g. www.blackdoginstitute.org.au/the-essential-network/burnout/[ID]). SAS CI360 will then be used to export all website analytics (e.g. time of access, pages visited) containing this unique record ID identifier and the Navigating Burnout pages visited during this session. These sessions will then be linked to individual participants’ data on REDCap using the associated URL record ID in order to objectively examine service usage.

In the active attention control, fortnightly emails containing links to digital self-care psychoeducation materials will be disseminated through Campaign Monitor – electronic direct mail software. Campaign Monitor automatically records when participants open an email, allowing for a measure of service usage in the active attention control arm.

### 2.12 Sample size

We will enrol 207 participants to complete the trial. This sample size is sufficient to examine the primary outcome using repeated measures ANOVA of whether the blended care version of the Navigating Burnout program leads to significantly lower burnout between baseline and 3 months post-intervention compared to self-guided treatment based on a power analysis (α = .05 and power = 0.80) using a conservative medium effect size of f = 0.25 between groups estimating a sample size of 53 per arm (WebPower). As there is limited literature on the effect of blended care treatments for burnout relative to self-guided digital psychotherapeutic programs, a medium effect size of f = 0.25 was chosen as a minimum clinically meaningful effect [28]. Accounting for a conservative estimate of 30% attrition based on previous research health professionals [19], 69 participants are required in each arm leading to a total sample size of 207.

### 2.13 Statistical methods

Study analyses will follow an intention-to-treat analysis plan using multiple imputation to account for missing data. To understand baseline variation and ensure experimental rigor, baseline scores on all psychosocial and occupational outcomes will be checked for between arm consistency using one-way analysis of variances (ANOVAs).

For the primary outcome (changes in OLBI burnout scores), between group variations (i.e., reductions in burnout between arms) will be examined using a 3 (blended, self-guided, active attention control) x 2 (baseline, 3 months post-treatment) mixed model ANCOVA controlling for gender, age, profession, and regionality and explored with pairwise comparisons. Identically formatted mixed model ANCOVAs will be used to analyse the secondary aims and outcomes of between arm and baseline to post-treatment and 3-month post-treatment differences in DASS (anxiety and depression), WSAS (psychosocial functioning), WHO-HPQ (workplace participation), and TIS-6 (attrition intention). The secondary aim and outcomes of comparing service acceptability between arms will be analysed using one-way ANOVAs.

If primary outcome data (i.e. OLBI at 3 months post-treatment) is unavailable for more than 5% of participants from the intention-to-treat population then exploratory and sensitivity analyses will be conducted. The following exploratory analyses of missing primary outcome data will be conducted to inform missingness categorisation and missing data handling: comparing proportion of attrition between arms, reasons for withdrawal, and baseline characteristics between those with and without the primary outcome. Best-worst and worst-best case sensitivity analyses will also be conducted alongside sensitivity analyses using last observation carried forward. Based on the results of these exploratory and sensitivity analyses, either complete case analysis or multiple imputation with 100 imputed data sets of the primary outcome will be conducted.

To examine the cost-effectiveness of blended (therapist-guided) Navigating Burnout, direct costs of the blended care arm will be quantified (computed as actual service delivery costs incurred by BDI) alongside indirect costs such as lost productivity of recipients due to use of the intervention (computed as average role wage multiplied by time taken out of work where sessions are attended during work hours, then annualised and adjusted for average FTE). Due to a lack of standardised and established estimates of the economic cost of burnout using commonly used measures, the benefits of blended Navigating Burnout will be compared against the value of improvements in occupational outcomes (WHO-HPQ; TIS-6), computed as average role wage multiplied by additional work engagement over the period of measurement, then annualised and adjusted for average FTE. Comparisons of these costs and benefits between treatment arms will provide an estimate of the net benefit and cost-effectiveness of blended Navigating Burnout. One-way and multi-way sensitivity analyses will be performed on assumptions in the model, such as cost estimates of lost productivity due to treatment and the relationship between turnover intention and actual turnover.

## 3 Ethics approval and trial registration

This RCT was approved by UNSW Human Research Ethics Committee (iRECS4616). All methods will be carried out in accordance with relevant guidelines and ethical approval granted by UNSW Human Research Ethics Committee. The RCT is registered with the Australian New Zealand Clinical Trials Registry (ACTRN12624000124538).

## 4 Results

It is anticipated that this trial will see improvement in burnout symptoms from baseline to 3 months post-treatment in both intervention arms (blended care and self-guided), with a more significant effect in the blended care arm. It is also anticipated that the trial will lead to improvements in the secondary aims and outcomes (anxiety and depression, psychosocial functioning, workforce participation, and workforce attrition intention) from baseline to 3 months post-treatment.

The active attention control is expected to be clinically inert (i.e. have no measurable effects on burnout outcomes, or the psychosocial and workforce secondary outcomes). However, the use of self-care materials by participants in the control arm may lead to some incidental improvements.

Navigating Burnout is expected to evidence high service acceptability among participants, and to be capable of implementation in a cost-effective manner.

## 5 Discussion

TEN is Australia's only blended care mental health support service designed by-and-for health professionals. While TEN has already shown strong promise in assisting health professionals to manage psychological distress, there is scope to improve and expand the service to better meet the needs of health professionals through programs designed to manage and reduce the symptoms of burnout among health professionals.

Through careful design to cater for the specific needs and demands placed on health professionals and considering their workplace culture, our Navigating Burnout digital program has the potential to address the dearth of effective treatment programs readily available to health professionals. This RCT will provide strong evidence that is expected to support not only the acceptability, but also the therapeutic efficacy and cost-effectiveness of Navigating Burnout in supporting doctors experiencing burnout across both self-guided and blended modalities. If found to be effective in this group, further evaluation in other health professional groups could also be considered.

If the results of this trial indicate high levels of acceptability and therapeutic benefit from blended care Navigating Burnout, the program will be incorporated into TEN's existing person-to-person blended care model to provide clinical psychologists treating health professionals with a guide to treating burnout using CBT and ACT. Alongside this, the digital Navigating Burnout program may act as an adjunct to therapy between person-to-person consultations.

With evidence of the cost-effectiveness of both blended care and self-guided Navigating Burnout, we expect the outcomes of this study to provide healthcare organisations across Australia with a valuable method to enhance the management of health professional well-being. Specifically, organisations would have the rationale and necessary clinical protocol to take a stepped approach to supporting doctors through burnout [1]: offering the digital Navigating Burnout tools as a self-guided format for people with mild or moderate symptoms of burnout or people reporting burnout who are waiting for person-to-person care from a mental health provider; and [2] partnering with their Employee Assistance Program or local mental health providers to offer the blended Navigating Burnout program.

## 6 Limitations

The self-guided digital Navigating Burnout program is currently publicly available through TEN without need for registration or an account. This decision followed the broad rationale behind TEN's development to reduce barriers to help-seeking and minimise health professionals' concerns around confidentiality. As such, there is a risk of contamination in the active attention control arm whereby participants could potentially access Navigating Burnout outside of the scope of the trial. To minimise the risk of contamination, references to TEN and Navigating Burnout will be omitted from recruitment materials and the PISCF.

Due to the nature of the funding for the trial, the participant population is limited to registered doctors. While this may limit the generalisability of findings to other health professionals, health professionals often share similar occupational environments and vulnerability to burnout. As such, there is a strong rationale for the applicability of findings to other health professional groups. A final limitation is that this intervention addresses the individual factors of burnout but does not directly address the more substantial systemic causes which, while beyond the scope of our study, urgently require greater attention.

## 7 Conclusion

In conclusion, there is a critical lack of established mental health services or treatment programs specifically for burnout in health professionals. The present trial has the potential to provide clear data on implementing digital cognitive-behavioural therapy for burnout into a blended care model, with such findings being essential to ensuring an effective, well-functioning, and sustainable health system for the benefit of the broader Australian community.

## CRediT authorship contribution statement

**M.J. Coleshill:** Writing – review & editing, Resources, Methodology, Funding acquisition, Writing – original draft, Project administration, Investigation, Conceptualization. **M.J. Black:** Resources, Funding acquisition, Writing – review & editing, Project administration, Conceptualization. **K. Luck:** Writing – original draft, Writing – review & editing. **K. Willis:** Writing – review & editing, Methodology, Resources. **N. Smallwood:** Resources, Writing – review & editing, Methodology. **H. Stephens:** Investigation, Methodology. **T. Gillings:** Investigation, Writing – review & editing. **L. Fraser:** Methodology. **M. Putland:** Resources. **L. Kampel:** Project administration, Resources. **A.M. Martin:** Project administration, Writing – review & editing. **N.F. Praharso:** Investigation, Writing – review & editing. **A.D. Joffe:** Investigation, Writing – review & editing. **S. Harvey:** Resources, Writing – review & editing. **P.A. Baldwin:** Resources, Methodology, Conceptualization, Writing – review & editing, Project administration, Funding acquisition.

## Data availability statement

As a clinical trial protocol, no data is presented as part of this publication.

## Funding sources

This project is funded by an 10.13039/501100020021Avant Foundation Grant.

## Declaration of competing interest

The authors declare the following financial interests/personal relationships which may be considered as potential competing interests: N/A.
